# Development and validation of a clinical prediction rule for chest wall syndrome in primary care

**DOI:** 10.1186/1471-2296-13-74

**Published:** 2012-08-06

**Authors:** Alexandre Ronga, Paul Vaucher, Jörg Haasenritter, Norbert Donner-Banzhoff, Stefan Bösner, François Verdon, Thomas Bischoff, Bernard Burnand, Bernard Favrat, Lilli Herzig

**Affiliations:** 1 Institute of General Medicine, University of Lausanne, Lausanne, Switzerland; 2 Department of Community Medicine and Primary care, University of Geneva, Geneva, Switzerland; 3 Department of General Practice/Family Medicine, University of Marburg, 35032, Marburg, Germany; 4 Institute of Social and Preventive Medicine, Lausanne University Hospital, Lausanne, Switzerland; 5 Department of Ambulatory Care and Community Medicine, University of Lausanne, Lausanne, Switzerland

**Keywords:** Chest pain, Primary care, Thoracic wall, Musculoskeletal system, Decision support techniques, Diagnosis

## Abstract

**Background:**

Chest wall syndrome (CWS), the main cause of chest pain in primary care practice, is most often an exclusion diagnosis. We developed and evaluated a clinical prediction rule for CWS.

**Methods:**

Data from a multicenter clinical cohort of consecutive primary care patients with chest pain were used (59 general practitioners, 672 patients). A final diagnosis was determined after 12 months of follow-up. We used the literature and bivariate analyses to identify candidate predictors, and multivariate logistic regression was used to develop a clinical prediction rule for CWS. We used data from a German cohort (n = 1212) for external validation.

**Results:**

From bivariate analyses, we identified six variables characterizing CWS: thoracic pain (neither retrosternal nor oppressive), stabbing, well localized pain, no history of coronary heart disease, absence of general practitioner’s concern, and pain reproducible by palpation. This last variable accounted for 2 points in the clinical prediction rule, the others for 1 point each; the total score ranged from 0 to 7 points. The area under the receiver operating characteristic (ROC) curve was 0.80 (95% confidence interval 0.76-0.83) in the derivation cohort (specificity: 89%; sensitivity: 45%; cut-off set at 6 points). Among all patients presenting CWS (n = 284), 71% (n = 201) had a pain reproducible by palpation and 45% (n = 127) were correctly diagnosed. For a subset (n = 43) of these correctly classified CWS patients, 65 additional investigations (30 electrocardiograms, 16 thoracic radiographies, 10 laboratory tests, eight specialist referrals, one thoracic computed tomography) had been performed to achieve diagnosis. False positives (n = 41) included three patients with stable angina (1.8% of all positives). External validation revealed the ROC curve to be 0.76 (95% confidence interval 0.73-0.79) with a sensitivity of 22% and a specificity of 93%.

**Conclusions:**

This CWS score offers a useful complement to the usual CWS exclusion diagnosing process. Indeed, for the 127 patients presenting CWS and correctly classified by our clinical prediction rule, 65 additional tests and exams could have been avoided. However, the reproduction of chest pain by palpation, the most important characteristic to diagnose CWS, is not pathognomonic.

## Background

When evaluating a patient with chest pain, the initial diagnostic step aims to rule out a life-threatening cause such as acute coronary syndrome or a pulmonary embolism [1]. However, the most common aetiology of chest pain in primary care practice is chest wall syndrome (CWS) [2], a benign source of chest pain localized to the anterior chest wall and caused by a musculoskeletal disorder [2,3]. Recent studies have shown that its incidence in primary care ranges from 20.4% to 46.6% [2,4-6].

CWS remains a diagnostic challenge [7]. Due to the absence of a consensus for diagnosing CWS, the diagnosis is usually obtained after the exclusion of other causes of chest pain, but this approach is time-consuming and requires important resources that could be directed elsewhere. Clinical signs related to CWS have been shown to be distinct from those of other more severe diagnoses [1], suggesting thata clinical prediction rule may help to identify patients with CWS. Although it is critical to exclude a life-threatening condition, reaching a diagnosis also seems to be very important for patients [8]. The need to develop non-invasive algorithms for primary care patients complaining of chest pain has been mentioned previously [9]. A literature review did not uncover a previously reported, validated clinical prediction rule for CWS [2,3,10-18], although a recent study described a four-point algorithm (localized muscle tension, stinging pain, pain reproducible by palpation and absence of cough) that can contribute to the diagnosis of CWS [10]. The aim of the present study was to develop and validate a clinical prediction rule for diagnosing CWS based on medical history and physical examination alone.

## Methods

### Design overview

We used data from the TOPIC (Thoracic Pain in Community) cohort, a multicentre cohort of primary care patients with chest pain, to develop a clinical prediction rule for CWS. We then analyzed data from a German study (the initial Marburg chest pain study, designed and conducted independently of the TOPIC cohort), a multicentre cohort of primary care patients with acute chest pain, to validate our rule. The original purpose of both studies was to investigate the characteristics of chest pain in primary care practice. Methods for both studies have been established previously and described (fully or partially) in various publications [2,6,10,19-30]. Later in the text, the TOPIC cohort will be referred as the “derivation cohort” and the German study as the “validation cohort”.

### Setting and participants: derivation cohort

General practitioners (GPs) in 58 independent medical offices and the medical residents of one university hospital outpatient department in Western Switzerland (counted as one additional practice) participated in the TOPIC study. All consecutive patients presenting with anterior chest pain (as a main or minor medical complaint) over a three-to-nine-week period (median length, five weeks) from March to May 2001 were included. Participating physicians had an average duration of experience in private practice of 12 years (range, 1–24 years). They received detailed information on the study and were trained to complete the forms during a meeting. The study protocol was approved by the Ethical Committee of the Canton of Vaud (Prot. 41/2000) [20].

### Setting and participants: validation cohort

Between October 2005 and July 2006, all attending patients with anterior chest pain (aged 35 years and over; n = 1249) were consecutively recruited to this study by 74 participating GPs in the state of Hesse, Germany. The recruitment period lasted 12 weeks for each practice. Patients were excluded when chest pain had lasted for one month or had already been noted by the primary care physician. The overall study protocol was approved by the Ethical Committee of the Faculty of Medicine, University of Marburg [6].

### Outcome and variables

CWS is defined as a benign cause of chest pain localized to the anterior chest wall and caused by a musculoskeletal disorder [2,3]. CWS is coded in the International Classification of Disease (ICD 10 R07.4) as “Anterior chest-wall pain not otherwise specified,” as well as in the International Classification of Primary Care (ICPC) under L04 as “Pain attributed to chest wall/pain attributed to musculoskeletal system” and under A11 as “Chest pain not otherwise specified” [31,32]. CWS includes fibromyalgia but excludes traumas, metastasis, and referred pain from the back. CWS encompasses various syndromes and clinical entities, such as costochondritis, costosternal syndrome, Tietze’s syndrome, chondrocostal pain, slipping rib syndrome, intercostal pain, left chondrocostal syndrome, left pectoral syndrome, and sternal syndrome [2].

A literature review was performed for two purposes. First, we wished to determine whether any clinical prediction rules for CWS had been generated for primary care. Bösner et al. developed a simple score containing four determinants [10], but it has not been validated in an external cohort. Second, we wished to identify relevant variables used to describe CWS for collection from patient histories and physical examinations (Table 1[2,3,10-18]). The following key words were used to search PubMed: "Chest Pain"[Mesh] AND ("Thoracic Wall"[Mesh] OR "Musculoskeletal System"[Mesh] OR "Musculoskeletal Diseases"[Mesh]) AND "Diagnosis"[Mesh]. In addition to the variables identified in the literature, those that appeared clinically relevant were selected for inclusion in this study.

**Table 1:** Variables indicating chest wall syndrome (ambiguous variables in bold)

**Medical history**		
*Thoracic pain described as*	Sharp/stinging	Trivial
	Aching	Lasting more than 5 min
	Not squeezing nor oppressive	Localized to one small area of the chest
	Pressure-like	Left or median left part of the chest wall
	Of varying intensity	Unilateral
*Relieving factors*	Decrease movement	Nitroglycerine don’t relieve
	Cessation of movement	Rest don’t relieve
	Change in position	Quiet breathing
	**Physical activity**	
*Associated/triggering factors*	**Exertion**	Physical activities that stress the upper body
	**Not exercise-induced**	**No consistency according to exercise**
	Certain activities	Unaccustomed physical activity
	At rest	Trunk movement/movement
	**Coughing**	Certain position
	**Absence of cough**	**Antecedent illness with coughing**
	Repeated minor trauma	Deep breathing
	History of rhumatoid arthritis	Psychic stress
**Physical examination**		
*Palpation*	Pain is reproducible	Chest wall tenderness
	Pain may not be reproducible	Localized muscle tension
	Paraspinal tenderness	
*Tests*	Horizontal flexion of arm	Spinal motion palpation restriction

### Data sources: derivation cohort

Information was recorded regarding GP activity, age, and experience, as well as patient basic characteristics, key past history, pain characteristics, and associated symptoms. The first part of the case report form (CRF) included 70 questions on history and clinical examination of chest pain. With the exception of age, all variables were dichotomized. Information about additional medical tests, the suspected diagnosis, and treatment decision was also recorded [20]. Follow-up data were obtained during additional consultations three and 12 months after the initial contact. CRFs included information on further examinations and laboratory assays, referrals to specialists, admissions to emergency wards, hospitalizations, and health events during the follow-up period. The initial suspected diagnosis was confirmed or modified during follow-up; we used the GP’s final diagnosis at 12 months as the reference diagnosis. When it was not possible to confirm the diagnosis, or if the diagnosis at 12 months was missing, the patient was contacted for further information through his GP. If the patient could not be contacted, the diagnosis at three months was retained. This method is not believed to be perfect, but is the best acceptable solution for studies in family practice settings [33]. The GP’s diagnosis was classified by the research team as CWS if it matched anything under this umbrella term as defined in the ICPC (see above, in section on outcome and variables).

### Data sources: validation cohort

As described previously [6], GPs took a standardized history and performed a physical examination according to a CRF. They also recorded their preliminary diagnoses, investigations, and management related to the patients’ chest pains. Patients were contacted by phone six weeks and six months after the index consultation. Study assistants blinded to the clinical data already recorded inquired about the course of the patients’ chest pain as well as treatments including hospitalization and drugs. Discharge letters from specialists and hospitals were requested from GPs.

### Protection against bias: derivation cohort

All completed forms were sent to the study coordination centre. A set of predefined criteria was used for data entry checks, and the GPs were contacted to resolve inconsistencies or to complete missing data. Double data entry was used to identify transcription errors. Data cleaning and validation was performed by a group of physicians experienced in research. When the diagnosis reported by the GP was not consistent throughout the follow-up year, the final diagnosis for chest pain was discussed and approved by a group of clinicians who were not aware of the aim of this study. Quality control of the reported diagnosis was done using patients up to date medical records at the GP’s office for a random ten percent sample of the included patients. No inconsistencies were identified.

### Protection against bias: validation cohort

As described previously [6], participating practices were recruited from a network of research practices associated with the University of Marburg Department of General Practice. The importance of recruiting every patient with chest pain irrespective of the presumed likelihood of ischemic heart disease was emphasized to the participating GPs. Practices were visited at 4-week intervals to check CRFs, recruitment logs, and compliance with study procedures. Random audits of the routine documentation of participating practices were implemented to identify cases of chest pain not included in the study. After six months, a reference panel consisting of one cardiologist, one GP, and one member of the research staff at the Department of Family Medicine reviewed the baseline and follow-up data of each patient. Analyzing all the information gathered during the follow-up period (results of further investigations, letters from specialists, hospital discharge reports, etc.), the panel decided on the most likely medical condition responsible for each patient’s chest pain at baseline.

### Statistical analysis

Using data from the derivation cohort alone, bivariate logistic regression analysis was performed to detect variables associated with CWS. Odds ratios (ORs) ≥ 2.0 were retained as potentially relevant for the multivariate analysis. To prevent overfitting related to colinearity, we explored the advantages of combining similar factors. For example, the variables “well localized pain (medical history)” and “well localized pain (physical examination)” were combined to create the variable “well localized pain (medical history and/or physical examination).” Variables were retained if their combination significantly improved the multivariate model compared to the use of either factor alone in the model (P-value of likelihood ratio test between models < 0.05). According to the regression coefficients in the multivariate analysis, weights (points) were attributed to each factor in order to build a score. The area under the receiver operating characteristic (ROC) curve was measured as an indicator of the discriminatory power of the score. We arbitrarily set the cut-off for specificity at ≥ 85%. Other researchers have used this cut-off for specificity [34].

The retained score was then applied to the validation cohort for external validation. Clinical signs that were not reported by the physician were considered absent by default. A second analysis was performed by multiple imputation for missing values of the key variable (pain reproducible by palpation). In the validation cohort, the best proxy for "pain well localized" was "localized muscle tension". The extent of pain from other tissues could not be taken into account. Area under the ROC curve, sensitivity, and specificity were calculated in both cohorts. All analyses were performed with StataCorp. 2009 Statistical Software (release 11.0, StataCorp, College Station, Texas, USA).

## Results

### Study population (derivation cohort)

**Figure 1:**
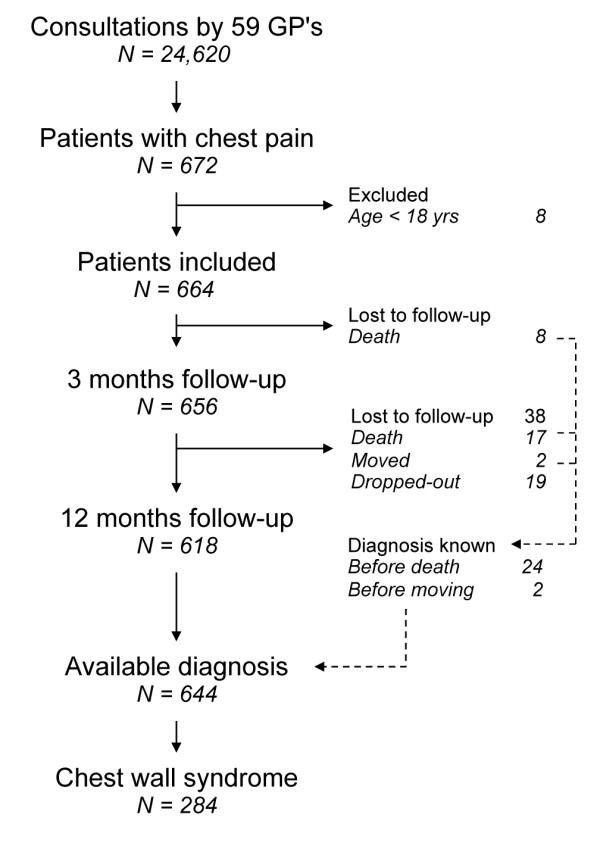
**Flow chart of derivation cohort: patient recruitment and follow-up data.** GPs = general practitioners.

A total of 664 patients with chest pain, aged 18 years and over, were included in the derivation cohort (22 patients were included and followed by the University Hospital Outpatient Department). Of these, 618 (93%) completed a 12-month follow-up with an available diagnosis. For the 46 cases with no formal diagnosis at 12 months, 25 died, two moved, and 19 had no diagnosis. However, diagnoses were available for 26 of these patients, with the diagnosis known before death or retained at the 3-month follow-up. Therefore, the population analyzed in this study consisted of 644 patients (Figure 1), 47.7% male, with mean age 55.4 years (range 18–95 years). CWS was diagnosed in 284 cases (Table 2). Chest pain was reproducible by palpation for 299/644 (46.4%) patients of the derivation cohort and for 201/284 (70.1%) of the CWS patients.

**Table 2:** Number, age and sex of derivation and validation cohorts: patients with CWS and whole study population at baseline

**Study**	**Baseline characteristics**	**CWS patients**	**Study population**
*Derivation cohort*	Number of patients (%)	284 (44.1)	644 (100.0)
	Mean age (range), years	50.5 (18–90)	55.4 (18–95)
	Sex : male patients, number (%)	136 (47.9)	307 (47.7)
*Validation cohort*	Number of patients (%)	565 (46.6)	1212 (100.0)
	Mean age (range), years	58 (35–90)	59 (35–93)
	Sex: male patients, number (%)	235 (41.6)	534 (44.1)

*CWS* chest wall syndrome.

### Building the clinical prediction rule

Comparing CWS patients with the remainder of the cohort, bivariate analyses identified 12 simple variables and three combined variables significantly associated with CWS (OR > 2.0) (Table 3). Only variables available for external validation (Table 3) were included in the regression model. A backward-stepwise method implemented to remove less-significant variables one by one resulted in the retention of six significant variables that were used to build the clinical prediction rule. The P-values, regression coefficients, and point attributions of these variables are shown in Table 4. The regression coefficients, which ranged from 0.5 to 1.6, were used to explore an accurate score model (points attributed for each variable ranging from 1 to 3, total score 10 points), but the accurate score model exhibited the same performance as the simplified model presented here.

**Table 3:** Odds of having chest wall syndrome (CWS) for all potential predictors and availability of each variable for external validation. Final variables retained for the clinical prediction rule are given in bold

**Simple variables**	**OR CWS vs. others (95% CI)**	**P-value**	**Available forexternal validation**
Trivial (history and physical examination)	9.11 (5.52-15.03)	<0.001	No
**Pain reproducible by palpation**	6.47 (4.58-9.15)	<0.001	Yes
**No history of coronary heart disease**	5.12 (2.88-9.12)	<0.001	Yes
Pain not retrosternal	5.08 (2.94-8.78)	<0.001	Yes
**Absence of general practitioner’s concern**	4.96 (2.79-8.84)	<0.001	Yes
Pain well localized (history)	2.65 (1.91-3.67)	<0.001	Yes
Pain superficial	2.48 (1.72-3.56)	<0.001	No
Pain well localized (physical examination)	2.46 (1.78-3.39)	<0.001	Yes
Pain not oppressive	2.37 (1.69-3.31)	<0.001	Yes
Pain related to position	2.13 (1.48-3.06)	<0.001	No
**Stabbing pain**	2.11 (1.38-3.24)	0.001	Yes
Pain related to movement	2.06 (1.42-3.01)	<0.001	Yes
Pain is not burning	1.62 (0.94-2.77)	0.081	Yes
Pain is not spread	1.52 (1.03-2.25)	0.037	No
Pain is not deep	1.30 (0.95-1.78)	0.100	No
Pain related to breathing	1.19 (0.83-1.71)	0.344	Yes
Absence of stressful stimulus	1.15 (0.72-1.82)	0.560	No
Intermittent pain	0.95 (0.69-1.29)	0.723	Yes
Absence of patient’s concern	0.92 (0.67-1.26)	0.613	Yes
Radiating pain	0.78 (0.51-1.20)	0.255	Yes
Pain is accompanied by cough	0.83 (0.56-1.24)	0.361	Yes
Pain related to cough	0.57 (0.37-0.89)	0.013	No
**Combined variables**^‡^			
**Pain neither retrosternal nor oppressive**	3.11 (2.24-4.33)	<0.001	Yes
**Pain well localized (history and/or physical examination)**	3.07 (2.17-4.35)	<0.001	Yes
Pain related to mechanical factors (position and/or movement)	2.56 (1.85-3.56)	<0.001	No
Pain is accompanied by digestive symptoms^§^	0.54 (0.35-0.85)	0.01	No

^§^Gastroesophageal reflux and/or odynodysphagia and/or dysphagia and/or epigastralgia.

^‡^Association of two simple variables.

*CWS* chest wall syndrome, *OR* odd ratio.

**Table 4:** **Multivariate analysis (pseudo R**^**2**^**: 0.2244): P-values, regression coefficients and number of points attributed to build the chest wall syndrome (CWS) clinical prediction rule**

**Variable**	**P-value**	**Regression coefficient**	**Number ofpoints attributed forrule**
Pain reproducible by palpation	<0.001	1.64	2
No history of coronary heart disease	<0.001	1.25	1
Absence of general practitioner’s concern	0.001	1.13	1
Pain neither retrosternal nor oppressive	0.017	0.48	1
Pain well localized (history and/or physical examination)	0.002	0.64	1
Stabbing pain	0.041	0.50	1
Total number of points attributable			7

*CWS* chest wall syndrome.

**Figure 2:**
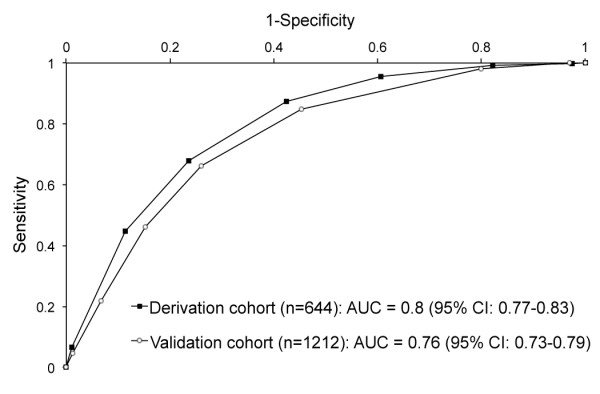
**Receiver operating characteristic curve for the chest wall syndrome clinical prediction rule in the derivation and validation cohorts.** AUC = area under curve.

**Figure 3:**
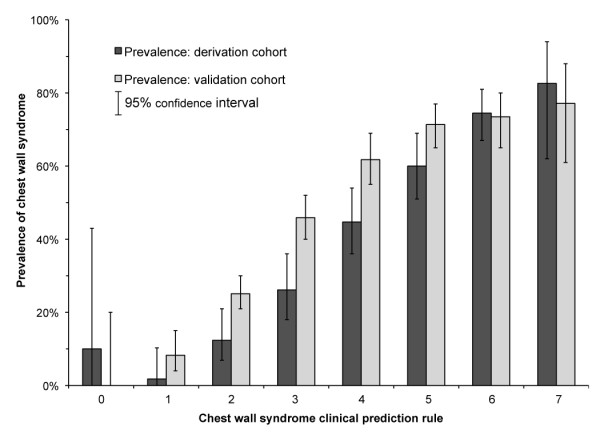
Observed prevalence of chest wall syndrome (CWS) in the derivation and validation cohorts for each clinical prediction rule value.

Given its stronger regression coefficient (1.64) the variable “pain reproducible with palpation” accounted for two points and the other variables for one point, for a total score ranging from 0 to 7 points. The area under the ROC curve was 0.8 (95% confidence interval 0.76-0.83; Figure 2). We set the cut-off point at 6 points, corresponding to a specificity of 88.6%. Application of this rule to the derivation cohort led to the correct classification of 127/284 (44.7%) patients with CWS. For 43 of these correctly classified CWS patients, 65 additional exams (30 electrocardiograms, 16 thoracic radiographies, 10 laboratory tests, eight specialist referrals, and one thoracic computed tomography) had been prescribed to reach the diagnosis. Figure 3 shows the observed prevalence of CWS for each score value. There were 41 false-positive patients, including three patients with stable angina (1.8% of all positives). Classification of each diagnostic subgroup by the CWS clinical prediction rule is detailed in Table 5.

**Table 5:** Classification of other diagnosis by the chest wall syndrome (CWS) clinical prediction rule

**Diagnostic groups**	**Classification by the CWS rule**	
	**False positive N = 41**	**True negative N = 476**
**Musculoskeletal (non-CWS)**	**18**	**23**
*Traumatic*	14	10
*Parietal metastasis*	0	7
*Post-thoracotomy*	2	4
*Arthritis/arthrosis*	2	2
**Cardiovascular**	**3**	**105**
*Stable angina*	3	72
*Unstable angina*	0	6
*Myocardial infarction*	0	4
*Pulmonary embolism*	0	2
*Arrythmia*	0	10
*Valvular disease*	0	2
*Cardiac insufficiency*	0	3
*Cardiomyopathy*	0	1
*Acute hypertension*	0	5
**Psychogenic**	**10**	**66**
*Anxious*	6	48
*Somatisation*	4	18
**Pulmonary**	**2**	**66**
*Infectious*	2	53
*Non-infectious*	0	13
**Digestive**	**3**	**52**
*Peptic affections*	1	46
*Non-peptic affections*	2	6
**Miscellaneous**	**5**	**7**
*Mastitis/mastalgia*	4	1
*Cutaneous abcess*	0	1
*Herpes zoster*	1	1
*Sarcoidosis*	0	2
*Chest wall keloid*	0	1
*Acute pyelonephritis*	0	1

### External validity

We accessed and used the Marburg database for external validation [6]. Diagnostic classification was possible for 1212 patients, including 565 diagnoses of CWS (46.6%). When applied to the validation cohort, the clinical prediction rule for CWS had an area under the ROC curve of 0.76 (95% confidence interval 0.73-0.79), 93.4% specificity, and 22.0% sensitivity (Figure 2), corresponding to a positive likelihood ratio of 3.3. Of all CWS patients, 124 were correctly classified (22% of total; Figure 3). There were 43 false positives, including three patients with stable angina (1.8% of all positives). No significant change was found after multiple imputation for missing values of the key variable “pain reproducible by palpation”.

## Discussion

We developed a clinical prediction rule for the diagnosis of CWS. The score ranges from 0 to 7 points, and the cut-off was set at 6 points to obtain specificity >85%. The rule contains the following six variables: thoracic pain (neither retrosternal nor oppressive), stabbing pain, well localized pain, no history of coronary heart disease, absence of GP concern, and pain reproducible by palpation (2 points). In the derivation cohort, the area under the curve was 0.8 and the specificity was 88.6% (319/360). The external validation showed 93% (604/647) specificity with an area under the curve of 0.76 (95% confidence interval 0.73-0.79). In addition, we observed that the reproduction of chest pain by palpation is not pathognomonic of CWS.

The strengths of our study include the large numbers of participating GPs and patients, allowing us to assume that our sample is fairly representative. We implemented a pragmatic strategy by consecutively including patients through their GP practice. We achieved an excellent follow-up at one year (96%), and more than half of the patients lost to follow-up had an available diagnosis. A key feature of our study was our external validation, which allowed us to confirm our results. However, our study suffers several limitations. First, delayed diagnosis was used as a reference in the absence of a panel of experts or an independent diagnostic process. It was not possible to perform additional tests systematically in all patients. Second, there is currently no consensus about the determinants or the designations of CWS in the literature. Third, there was no specific calibration for the variable “pain reproducible with palpation,” which may have induced differences in individual interpretations. Fourth, some variables were not available for external validation, and although they were relevant to the bivariate analyses, they could not be included in the development of the clinical prediction rule. Fifth, the two cohorts showed design differences, what could weaken their comparison. Only patients with acute chest pain were included in the validation cohort; in addition, the reference diagnosis was made by means of a telephoneinterview of the patient in the validation cohort. The TOPIC study was not originally designed for the development of a clinical prediction rule for CWS; if it had been, then other diagnostic criteria may have been explored.

Our requirement that the prediction rule be highly specific leads, unsurprisingly, to a relatively low rate of patients with CWS correctly classified in the derivation cohort (127/284 = 45%). A lower rate (124/565 = 22%) in the validation cohort can be partly explained by design differences and semantic limits. In the derivation cohort, the 41 false positives were not life-threatening cases; most of these cases were easily identifiable and were associated with a plausible pathophysiological mechanism for chest pain such as trauma or thoracotomy. Interestingly, parietal metastases were not classified as false positives. However, our score misclassified three patients (1.8% of all positives cases) with stable angina as CWS. For these three patients, the GPs had noted symptoms and signs for CWS as well as for cardiovascular disease, and we therefore suspect the co-existence of both diagnoses for these three patients. Therefore, patients with a positive CWS clinical prediction rule in the presence of other signs of chest pain must be carefully examined and monitored for the final diagnosis and evolution of chest pain.

In the literature, we found no consensus about the classification or the determinants of CWS, and the pertinence of some variables is ambiguous. This lack of clarity had already been identified [2,3,35,36]. Our study provides a clear definition of CWS as well as a diagnostic tool. Although another score has been previously described [10], we have presented a validated tool. Another important difference between these two works is the variable “absence of cough;” whereas this variable is not relevant in our cohort, it was included in the four-point score developed by Bösner et al. [10]. However, since this variable is interpreted in various ways in the literature, we believe that it is not optimal in the diagnosis of CWS. Finally, the reproducibility of chest pain by palpation is addressed repeatedly in the literature. According to our clinical prediction rule, this variableis necessary for the diagnosis of CWS. Although this reproducibility was previously thought to be the strongest evidence of CWS [4], more recent work has demonstrated that it is not pathognomonic of CWS [3,37,38], a finding we have confirmed.

Universal consensus requiresthe exclusion of a potentially life-threatening cause of chest pain in the emergency department and in primary care practice [1,39], and patients with CWS are referred to their GPs for further investigation. There is a low prevalence of patients presenting CWS in the emergency department [5], and this disease is not followed up there. However, in addition to an emergency department triage approach, GPs must establish the diagnosis, and will treat and follow their patients regardless of the final diagnosis. Our clinical prediction rule will aid this process. Further studies may be necessary to verify the effectiveness of our rule and to improve the management of CWS.

## Conclusion

In conclusion, CWS is a frequent diagnosis in primary care that is well known by general practitioners but has been insufficiently studied in the literature. Our proposed clinical prediction rule constitutes a tool that can be used in addition to the usual process of diagnosing CWS by elimination. Moreover, because its high specificity, a high positive score may help clinicians to avoid ordering additional tests. For instance, using this tool, 65additional exams for 127 patients could have been avoided in the derivation cohort. We propose that our clinical prediction rule should be included in the clinical diagnostic reasoning of physicians encountering a patient with chest pain in primary care, keeping in mind that it does not exclude the presence of concomitant diagnoses.

## Abbreviations

AUC, Area under the curve; CRF, Case report form; CWS, Chest wall syndrome; GP, General practitioner; OR, Odd ratio; R^2^, Coefficient of determination; ROC, Receiver operating characteristic; 95%CI, 95% confidence interval.

## Competing interests

The authors declare that they have no competing interests.

## Authors’ contributions

FV and LH conceived the study, participated in patient inclusion, were the principle investigators, and critically revised the manuscript. BB and BF participated in the design of the study and critically revised the manuscript. PV designed the statistical analysis, and participated in drafting the manuscript. AR performed the statistical analysis and wrote the manuscript. TB participated in patient inclusion and critically revised the manuscript. JH, NDB and SB participated in the statistical analyses of the external validation and critically revised the manuscript. All authors accepted the manuscript after reading.

## Pre-publication history

The pre-publication history for this paper can be accessed here:

http://www.biomedcentral.com/1471-2296/13/74/prepub
